# Bringing cost-effectiveness models to life: AI Replication of a published model of venlafaxine treatment in major depressive disorder using a large language model (LLM)

**DOI:** 10.1192/j.eurpsy.2026.11693

**Published:** 2026-07-31

**Authors:** N. Hawkins, R. Thompson, K. Subramaniam, P. Purushottamahanti

**Affiliations:** 1 Visible Analytics, Oxford; 2 Health Economics and Outcomes Research, Viatris, London, United Kingdom; 3 Global Medical Affairs, Viatris, Auckland, New Zealand; 4 Global Medical Affairs, Viatris, Bangalore, India

## Abstract

**Introduction:**

Cost-effectiveness (CE) models are an important decision-making aid when making re-imbursement decisions within resource constrained health systems. However, unlike the results of clinical trials, the findings from cost-effectiveness are highly context dependent. They may depend on choice of comparators, local cost estimates, and current estimates of effectiveness and patient utilities.

**Objectives:**

The objective of this project was to investigate whether a fully working model adaptable to a local context could be developed using an LLM based on a published manuscript.

**Methods:**

We attempted to develop a working version of the CE model of venlafaxine treatment described by Sobocki et al. (Sobocki et al. Int J Clin Pract, April 2008, 62, 4, 623–632) in the R statistical language using ChatGPT 5.0 based on simple prompts and a PDF copy of the manuscript.

**Results:**

The prompt “please develop R code for the model described in this paper” produced functioning R code that did not require modification. However, the published results were not replicated. The prompt “which parameters are not explicitly stated in the paper” identified the placebo relapse rate, background mortality, and venlafaxine acquisition cost as not fully reported and identified those were most influential. In addition, ChatGPT identified the key parameters offered to ‘back-fit’ plausible values for these parameters. Using these estimates the model results matched published results. The model was then validated by code inspection and independent replication in R.

**Conclusions:**

ChatGPT 5 successfully replicated the model based on simple prompts and minimal iteration. It was also able to identify inadequately specified model parameters and, significantly, able to conduct a calibration exercise to estimate these. The ability of ChatGPT 5 to replicate published cost-effectiveness models is much improved on previous versions only requiring simply prompts and, in this case, producing code that ran ‘first time’. This model can be used to estimate the cost effectiveness of venlafaxine based on current costs and local contexts that may enable clinicians to make more informed clinical decisions. Will ChatGPT 5 ‘democratise’ cost-effectiveness analysis?

**Disclosure of Interest:**

N. Hawkins: None Declared, R. Thompson Employee of: Viatris, K. Subramaniam Employee of: Viatris, P. Purushottamahanti Employee of: Viatris

